# Response of Phyllosphere and Rhizosphere Microbial Communities to Salt Stress of *Tamarix chinensis*

**DOI:** 10.3390/plants13081091

**Published:** 2024-04-13

**Authors:** Xuan Qu, Yaqing Pan, Peiqin Wang, Lele Ran, Guifei Qin, Qunfang Li, Peng Kang

**Affiliations:** 1 School of Biological Science and Engineering, North Minzu University, Yinchuan 750021, China; quxuan99@outlook.com (X.Q.); w13036751168@outlook.com (P.W.); rjx1429364135@outlook.com (L.R.); qingf1234@outlook.com (G.Q.); lqf2507418297@outlook.com (Q.L.); 2 Shapotou Desert Research and Experiment Station, Northwest Institute of Eco-Environment and Resources, Chinese Academy of Sciences, Lanzhou 730000, China; 3 Innovation Team for Genetic Improvement of Economic Forests, North Minzu University, Yinchuan 750021, China

**Keywords:** halophyte, salt secretion, rhizosphere microbiome, phyllosphere microbiome, functional groups

## Abstract

As carriers of direct contact between plants and the atmospheric environment, the microbiomes of phyllosphere microorganisms are increasingly recognized as an important area of study. Salt secretion triggered by salt-secreting halophytes elicits changes in the community structure and functions of phyllosphere microorganisms, and often provides positive feedback to the individual plant/community environment. In this study, the contents of Na^+^ and K^+^ in the rhizosphere, plant and phyllosphere of *Tamarix chinensis* were increased under 200 mmol/L NaCl stress. The increase in electrical conductivity, Na^+^ and K^+^ in the phyllosphere not only decreased the diversity of bacterial and fungal communities, but also decreased the relative abundance of Actinobacteriota and Basidiomycota. Influenced by electrical conductivity and Na^+^, the bacteria–fungus co-occurrence network under salt stress has higher complexity. Changes in the structure of the phyllosphere microbial community further resulted in a significant increase in the relative abundance of the bacterial energy source and fungal pathotrophic groups. The relative abundance of Actinobacteriota and Acidobacteriota in rhizosphere showed a decreasing trend under salt stress, while the complexity of the rhizosphere co-occurrence network was higher than that of the control. In addition, the relative abundances of functional groups of rhizosphere bacteria in the carbon cycle and phosphorus cycle increased significantly under stress, and were significantly correlated with electrical conductivity and Na^+^. This study investigated the effects of salinity on the structure and physicochemical properties of phyllosphere and rhizosphere microbial communities of halophytes, and highlights the role of phyllosphere microbes as ecological indicators in plant responses to stressful environments.

## 1. Introduction

Global climate change, coupled with human activities, has aggravated soil desertification and salinization [1,2]. In this context, the use of halophytes to improve and manage saline lands has been effective [3,4]. Halophytes can not only survive in a high-salt environment, but they also play an active role in soil improvement through the characteristics of salt secretion and salt accumulation. As a “bridge” along the plant–soil continuum, plant rhizosphere microorganisms also have positive significance in enhancing plant nutrient uptake and biogeochemical cycling in soil under stress [5,6]. In high-salt environments, halophytes alter the survival strategies of rhizosphere microorganisms through the release of root exudates (organic acids, growth hormones, etc.) [7]. Meanwhile, the rhizosphere of halophytes can also be adapted to oligotrophic, high-salt environments by the recruitment of specific microbial communities [8]. As plants provide space for the growth and reproduction of rhizosphere microorganisms, the competition for limited resources amongst halophyte rhizosphere microorganisms changes the structure and function of the community in a high-salinity environment [9]. At present, the mechanism of rhizosphere bacterial–fungal interactions of halophytes in response to high-salt habitats deserves further study.

Halophytes can be divided into salt-rejecting and salt-secreting [10]. Salt-secreting halophytes can secrete large amounts of toxic ions that accumulate in the leaves through salt glands or salt bladders, maintaining the ionic homeostasis of leaf cells while adapting to saline environments [11,12]. The phyllosphere is a unique habitat for microbial communities, and the changes in the microbiome are closely related to the physiological characteristics of the host [13,14]. Plant leaf structure shapes the habitat of colonizing microorganisms [13]. For example, leaf trichoid structure and epidermal cells encourage the recruitment and aggregation of microorganisms [15]. Meanwhile, the sugars, inorganic salts, and water exuded by leaf cells accumulate in these grooves, providing resources for the colonizing microorganisms [16]. Accordingly, the cone-shaped salt glands affect the living spaces and strategies of phyllosphere microbial colonizers when a large quantity of salt is released in salt secretion events. However, little is known about the effects of salt secretion on the function of phyllosphere microbial communities.

In addition, when salt scatters on the leaf surface and fills the grooves, the space in which the phyllosphere colonizers thrive is reduced, perhaps through competition to occupy ecological niches and increase access to limited resources [17,18]. Indeed, several studies on phyllosphere microbial communities have noted that rainfall reduces the bacterial spectrum of leaf microbial communities [19,20]. Furthermore, environmental factors such as wind, humidity, or ultraviolet radiation reduce the phyllosphere fungal diversity [21]. Interestingly, classes such as Methylophilaceae, Pseudomonadaceae, and Sphingomonadaceae can compete for survival in stressful environments and contribute to stress tolerance in host plants by producing phytohormones, forming biofilms, and participating in the synthesis of extracellular polysaccharides [22]. Therefore, in extreme environments, the competition for ecological niches among phyllosphere colonizers depends on their survival strategy [23,24], the selection of which is still not well-understood. 

As mentioned above, the structures and changes of phyllosphere microbial communities on salt-secreting halophytes have received little attention. As a desert plant with salt glands, *Tamarix chinensis* has a pronounced ecological effect, and its phyllosphere harbors a unique microbial community adapted to extreme environments [25,26]. In China, *T. chinensis* is often used as the principal plant in the restoration of saline–alkaline land. It performs varied ecological functions, thereby playing an important role in maintaining biogeochemical cycles while stabilizing factors such as soil pH, electric conductivity, and cations [27,28]. However, the response characteristics of the phyllosphere colonizers of the salt-secreting halophyte *T. chinensis* to salt secretion remain unclear. Therefore, in this study, the compositional, structural and functional changes of phyllosphere and rhizosphere microbial communities of *T. chinensis* in response to salt stress were comprehensively analyzed using field observations as well as controlled indoor experiments. The following scientific issues are to be addressed: (1) the characterization of phyllosphere and rhizosphere microbial communities of *T. chinensis* in response to salt stress; (2) the changes in phyllosphere and rhizosphere microbial functional groups of *T. chinensis* under salt stress.

## 2. Results

### 2.1. Physicochemical Properties in Phyllosphere and Rhizosphere of T. chinensis under Salt Stress

**Figure 1:**
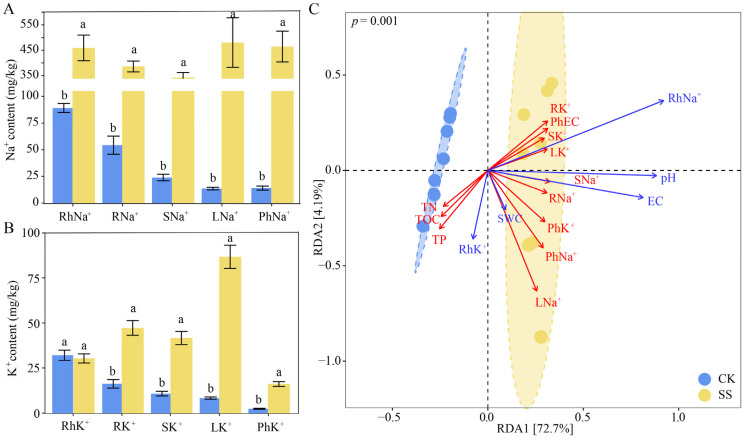
Na^+^ (**A**) and K^+^ (**B**) contents in rhizosphere, tissues and phyllosphere of *T. chinensis* under salt stress; RDA analysis of physicochemical properties (**C**). CK: control group. SS: salt stress group. RhNa^+^: rhizosphere Na^+^ content. RNa^+^: root Na^+^ content. SNa^+^: stem Na^+^ content. LNa^+^: leaf Na^+^ content. PhNa^+^: phyllosphere Na^+^ content. RhK^+^: rhizosphere K^+^ content. RK^+^: root K^+^ content. SK^+^: stem K^+^ content. LK^+^: leaf K^+^ content. PhK^+^: phyllosphere K^+^ content. Different lowercase letters indicate differences between groups.

Soil electric conductivity (EC) and Na^+^ and K^+^ contents increased significantly under salt stress, but had no significant effect on soil water content (SWC) and pH (*p* < 0.05). Meanwhile, soil total organic carbon (TOC), total nitrogen (TN) and total phosphorus (TP) contents were reduced to different degrees under salt stress (18.25%, 25.19% and 16.36%) (Table 1). Furthermore, the contents of Na^+^ and K^+^ in the root, stem, and leaves of *T. chinensis* were significantly higher under salt stress than in the control (CK) (*p* < 0.05). The contents of phyllosphere EC, Na^+^ and K^+^ under salt stress were also significantly higher than in the control, by 2.78, 32.04 and 5.61 times, respectively (*p* < 0.05) (Figure 1A,B). Redundancy analysis (72.7% and 4.19% explained) indicated that soil EC and Na^+^ content had a positive effect on Na^+^ and K^+^ content in different tissues, as well as phyllosphere, while soil TOC, TN and TP contents were affected by Na^+^ content (*p* = 0.001) (Figure 1C).

### 2.2. Microbial Diversity in Phyllosphere and Rhizosphere of T. chinensis under Salt Stress

**Figure 2:**
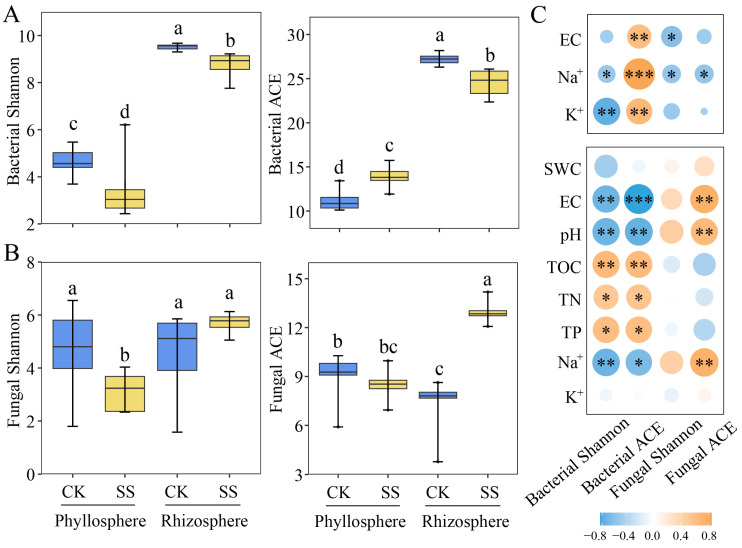
Alpha diversity of bacteria (**A**) and fungi (**B**) in the phyllosphere and rhizosphere and (**C**) the correlation with physicochemical properties of *T. chinensis* under salt stress (* *p* < 0.05; ** *p* < 0.01; *** *p* < 0.001). Different lowercase letters indicate differences between groups.

Shannon index is usually used to represent the diversity of microbial community, and the abundance-based coverage estimator (ACE) represents the richness of the microbial community, which is an important parameter of microbial community alpha diversity. The Shannon indices of phyllosphere and rhizosphere bacteria were decreased under salt stress, but the abundance-based coverage estimators (ACE) indices of phyllosphere bacteria were increased (Figure 2A). The Shannon and ACE indices of phyllosphere fungi were decreased, while the ACE index of rhizosphere fungi increased under salt stress (Figure 2B). Spearman’s correlation found that the Shannon index was negatively correlated with Na^+^ and K^+^, while ACE was positively correlated with EC, Na^+^ and K^+^ in phyllosphere bacteria. In addition, rhizosphere soil EC, pH and Na^+^ were negatively correlated with bacterial Shannon and ACE indices, while TOC, TN and TP were positively correlated with bacterial Shannon and ACE indices (Figure 2C). It can be seen that the effects of salt inputs on soil physicochemical properties altered the Shannon and ACE indexes of rhizosphere bacteria and fungi. At the same time, the secretion and accumulation of phyllosphere in *T. chinensis* further altered the Shannon and ACE indexes of phyllosphere bacteria and fungi. 

### 2.3. Microbial Community Structure in Phyllosphere and Rhizosphere of T. chinensis under Salt Stress

**Figure 3:**
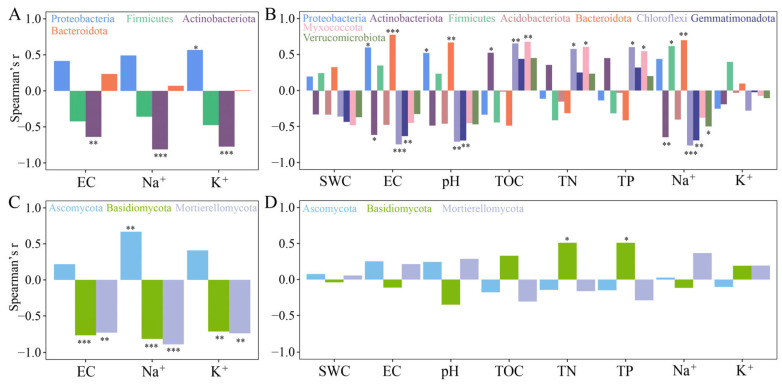
Correlation of bacterial and fungal phyla (relative abundance > 1%) with leaf physicochemical properties (**A**,**B**) and rhizosphere physicochemical properties (**C**,**D**) of *T. chinensis* under salt stress (* *p* < 0.05; ** *p* < 0.01; *** *p* < 0.001).

Proteobacteria (66.43–80.02%), Actinobacteria (15.37–2.56%), Firmicutes (14.47–12.25%) and Bacteroidota (1.83–3.66%) were the dominant bacteria phyla in the phyllosphere of *T. chinensis* under salt stress (Figure S2A). Salt stress significantly reduced the relative abundance of Actinobacteriota in phyllosphere (Table S1), and Spearman’s correlation analysis revealed a significant negative correlation between EC, Na^+^, and K^+^ in the phyllosphere (Figure 3A). In the rhizosphere, Proteobacteria (18.99–25.97%), Actinobacteriota (16.6–8.66%), Firmicutes (7.64–15.45%), Acidobacteriota (14.12–8.59%), Bacteroidota (1.78–10.81%), Chloroflexi (5.9–3.13%), Gemmatimonadota (1.84–0.8%), Myxococcota (1.52–0.97%) and Verrucomicrobiota (1.19–0.87%) were the dominant bacterial phyla in the rhizosphere of *T. chinensis* (Figure S2B). Salt stress significantly decreased the relative abundance of Actinobacteriota and Acidobacteriota and increased the relative abundance of Bacteroidota in the rhizosphere (Table S1). Spearman’s correlation analysis revealed that Bacteroidota was significantly and positively correlated with EC and pH. It is worth noting that the relative abundances of Chloroflexi and Gemmatimonadota showed no significant differences in the rhizosphere regardless of control or salt stress, but showed a significant negative correlation with EC, pH and Na^+^ (Figure 3B).

Among the fungal communities, Ascomycota (56.4–75.0%), Basidiomycota (17.69–1.04%) and Mortierellomycota (9.28–0.11%) were the most dominant phyla in the phyllosphere of *T. chinensis* (Figure S2C). Salt stress increased the relative abundance of Ascomycota while decreasing the relative abundance of Basidiomycota and Mortierellomycota (Table S2), while Basidiomycota and Mortierellomycota showed a significant negative correlation with EC, Na^+^ and K^+^ (Figure 3C). In the rhizosphere, Ascomycota (62.44–66.73%), Basidiomycota (9.01–4.87) and Mortierellomycota (5.24–8.23%) were not significantly affected by salt stress, and only Basidiomycota showed a significant positive correlation with TN and TP (Figure S2D and Figure 3D).

### 2.4. Microbial Co-Occurrence Network in Phyllosphere and Rhizosphere of T. chinensis under Salt Stress

**Figure 4:**
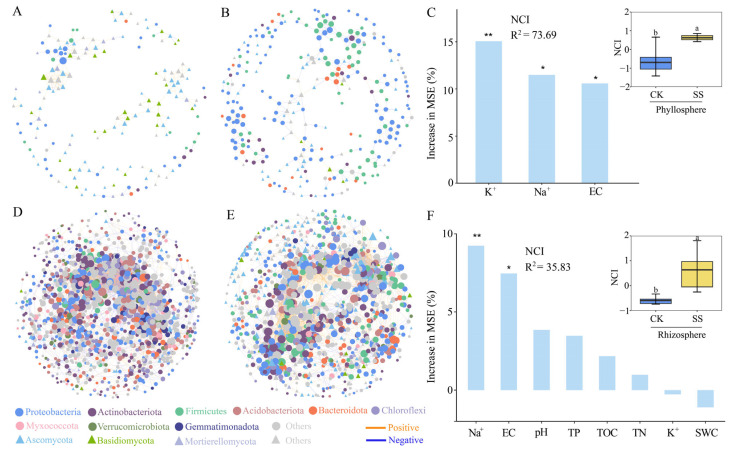
Co-occurrence network of phyllosphere (**A**,**B**) and rhizosphere (**D**,**E**), and network complexity indices (NCI) of phyllosphere (**C**) and rhizosphere (**F**), along with the random forest analysis of the physicochemical properties of *T. chinensis* under salt stress (* *p* < 0.05; ** *p* < 0.01).Different lowercase letters indicate differences between groups.

Co-occurrence network analysis of phyllosphere showed that SS treatment yielded more nodes (175–280) and edges (175–280). Meanwhile, the average path length, graph diameter and betweenness centralization were also higher than in CK (Table 2). SS treatment significantly increased the network complexity index (NCI) of the phyllosphere co-occurrence network, and random forest analysis found that K^+^, Na^+^, and EC had high explanatory effects for the NCI (Figure 4C). In contrast, the number of nodes (1384–1150) and edges (7531–6394) decreased under SS treatment in the *T. chinensis* rhizosphere co-occurrence network (Figure 4D,E) (Table 2), but the NCI increased and was significantly higher than that of CK, while random forest analysis revealed that Na^+^ and EC had a high explanatory rate for NCI (Figure 4F).

### 2.5. Microbial Functional Groups in Phyllosphere and Rhizosphere of T. chinensis under Salt Stress 

FAPROTAX functional predictions revealed that SS treatment significantly increased the relative abundance of energy source groups (20.44–37.17%) (for example, chemoheterotrophy and photoheterotrophy) in phyllosphere of *T. chinensis*. At the same time, the relative abundance of carbon cycle (5.22–11.39%) and sulfur cycle (0.14–1.27%) groups of rhizosphere bacteria increased under SS treatment (Table 3). FUNGuild functional predictions found differences between saprotrophic and pathotrophic groups of phyllosphere fungi, with SS treatment decreasing the relative abundance of saprotrophic groups (33.21–3.34%) and increasing the relative abundance of pathotrophic groups (36.84–70.68%). SS treatment did not have a significant effect on the functional groups of rhizosphere fungi (Table 4).

**Figure 5:**
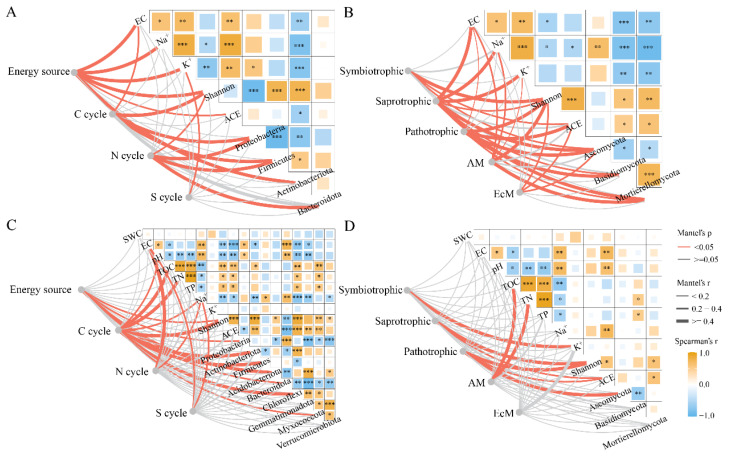
Mantel’s test for functional bacteria and fungi with physicochemical properties and differential phyla in phyllosphere and rhizosphere of *T. chinensis* under salt stress. (**A**) Phyllosphere bacteria; (**B**) rhizosphere bacteria; (**C**) phyllosphere fungi; (**D**) rhizosphere fungi. (* *p* < 0.05; ** *p* < 0.01; *** *p* < 0.001).

In the phyllosphere, EC, Na^+^, K^+^, bacterial diversity and dominant phyla were all significantly correlated with phyllosphere bacterial functional groups, while the correlations between EC, Na^+^, K^+^, fungal diversity, dominant phyla and fungal functional groups were also significant. In the rhizosphere, energy source and C cycle groups were significantly affected by the rhizosphere soil physicochemical properties, bacterial diversity and dominant phyla, while nitrogen cycle and sulfur cycle were more closely related to EC and Na^+^. There were more pronounced correlations between symbiotrophic and K^+^, symbiotrophic and Shannon index, and pathotrophic and Ascomycota. In addition, AM was more significantly affected by TOC and TN (Figure 5).

## 3. Discussion

### 3.1. Rhizosphere Microorganisms Respond to Salt Stress through Niche Competition and Adaptation Strategies

*T. chinensis* plays an important ecological role in improving saline–alkaline land in northern China [28,29]. We found that with increased soil salinity, roots, stems and leaves of *T. chinensis* accumulated a larger amount of Na^+^ than that observed in the control; meanwhile, the K^+^ content also increased significantly (Figure 2). The higher accumulation of Na^+^ and K^+^ in plant tissues indicated that key genes mediating ion regionalization and encoding or regulating related transcription factors play an important role in halophytes’ resistance to salt stress [30,31]. On the contrary, K^+^ accumulation helps plants maintain a high K^+^/Na^+^ ratio, which may play an important role in *T. chinensis* resistance to salt stress. Similar results were reported by studies on salt-secreting halophytes *Limonium bicolor* and *Atriplex canescens* [12,32]. 

In our study, the increase in soil salinity not only enhanced the Na^+^ and K^+^ contents within the tissues and between the phyllosphere of *T. chinensis*, but it also decreased the contents of soil TOC, TN, and TP (Table 1). Therefore, we hypothesize that salt stress alters the rhizosphere microbial community, thereby affecting the biogeochemical cycles of the functional flora [33,34]. Consistent with our findings, salt stress decreased the alpha diversity index of *T. chinensis* rhizosphere (Figure 2). A recent study [35] indicated that soil salinity negatively impacts effective soil nutrient utilization. On the other hand, it is plausible that appropriate salinity (leading to stress but not death) improves halophyte growth in some ways, such as improving the utilization of nitrogen or phosphorus [12,36]. In this study, the salt treatment was ended when the salt secretion of the phyllosphere was visible but the plants were still not killed by the stress. Therefore, the changes in soil TOC, TN, and TP contents under salt stress may be influenced by a combination of the above two factors.

Proteobacteria and Actinobacteriota are the dominant bacteria of the *T. chinensis* rhizosphere (Figure 3). Changes in soil moisture, pH, salinity, and nutrients significantly affected the rhizosphere microbial community structure of *T. chinensis*, consistently with previous studies [37]. Among fungi, Ascomycota and Basidiomycota were the dominant phyla in the rhizosphere of *T. chinensis*, but their composition was also affected by salt stress [35]. In addition, the network relationships among microorganisms can also reflect the occupation of ecological niches by each OTU [38]. In our study, salt stress increased the positive correlation among OTUs of rhizosphere bacteria of *T. chinensis*, which was speculated to be the result of cooperative resistance to saline habitats among OTUs [33]. 

### 3.2. Salt Secretion Influences Microbial Occupation of the Phyllosphere Niche

The phyllosphere EC, Na^+^, and K^+^ contents of *T. chinensis* were significantly higher than in the control after salt treatment, which is consistent with previous results [39]. This shows that halophytes resist salt stress by secreting salt under salt treatment conditions without harming plant cytoplasm (NaCl concentration: 100–200 mmol/L). Indeed, Pan et al. [12] reported that *Atriplex canescens* secreted salt from salt bladders in response to treatment with 400 mmol/L NaCl, which might be related to the salt-secretion adaptation of the plant. In *T. chinensis*, excess salt is released into the environment through salt glands, which is an important survival mechanism for *T. chinensis* to resist excess salinity in its environments [11]. 

The composition of the phyllosphere microbial community of *T. chinensis* is reportedly dominated by Proteobacteria, Bacteroidetes, Firmicutes and Acidobacteria (Figure S2); consistently, Finkel et al. [40] showed that Proteobacteria, Bacteroidetes and Firmicutes were the dominant bacterial phyla in the phyllosphere of *T. chinensis*. From a niche-assemblage perspective, the diversity of phyllosphere microbial communities is maintained by partitioning organisms into specific ecological niches and allowing several species to coexist [40]. Salt secretion by *T. chinensis* reduced the diversity of bacterial and fungal communities in phyllosphere, but increased bacterial community richness (Figure 2). We hypothesized that salt secretion exerts a stimulating effect, which induces a coordinated response in the phyllosphere colonizers to improve community structure stability [41,42]. Most research studies on microbial priming effects have focused on plant–soil feedback. However, our study was the first one to focus on the effects of salt secretion on plant phyllosphere. Environmental selection is an important factor affecting microbial community structure and interrelationships [7], and in our study, the structure of the phyllosphere microbial community was significantly changed by salt secretion, especially in terms of the microorganisms’ competition for ecological niches. 

### 3.3. The Functional Groups of Microorganisms in the Phyllosphere and Rhizosphere of T. chinensis Were Closely Related to Environmental Changes 

Salt stress affects potential functions such as microbial metabolism and material cycling in plant rhizosphere soils [43]. In this study, salt stress increased the relative abundance of rhizosphere bacterial carbon and sulfur cycle groups in *T. chinensis*, and it has been shown that changes in the carbon cycle group are more pronounced in the rhizosphere in response to salinity stress [44]. This may be caused by the effects of salt stress on the plant root system, resulting in an increase in plant root residues and changes in soil structure [45]. It has also been reported that the increase in the abundance of genes related to rhizosphere carbon and sulfur cycling under salt stress may mitigate the negative effects of salt on plants [46]. Through Mantel’s analysis, we further found that EC, pH, Na^+^ and K^+^ were closely related to elemental cycling groups, not only reflecting the close relationship between the ecological functions of rhizosphere bacteria and environmental factors, but also further demonstrating the important role of plant fitness in stress response [47].

Salt stress also increased the relative abundance of phyllosphere energy source groups. This may be because the large amounts of inorganic ions and organic matter released from salt gland cells provided a rich resource for colonization. Meanwhile, the nitrogen fixation of the phyllosphere microbiome is an important pathway for leaf nutrient acquisition [48,49,50,51]. In our study, the phyllosphere carbon and nitrogen cycle groups of *T. chinensis* were found to be closely related to environmental changes. It has been further confirmed that plant phyllosphere bacteria play an important role in nitrification, methylation and anoxic photosynthesis [52,53]. In addition, we confirmed that phyllosphere microorganisms, such as certain types of Proteobacteria members, enhance phosphorus access by dissolving phosphate [18,54]. We also noted that the relative abundance of these taxa was reduced by salt secretion. Phyllosphere fungi perform a wide range of metabolic functions and are involved in the early decomposition of leaf litter and the cycling of nutrients [55]. In our study, salt secretion decreased the relative abundance of saprotrophic groups.

## 4. Materials and Methods

### 4.1. Plant Materials and Treatments

The experimental materials were collected from a *T. chinensis* plantation in Yanchi County, Ningxia (107°18′ E, 37°39′ N). In April 2021, the middle stems of *T. chinensis* were cut at about 80–100 cm from the ground and brought to the laboratory (Figure S1A). The cuttings were propagated according to the method of Zhang et al. [30]. When the seedlings developed adventitious roots and young leaves, they were moved into pots (32 cm (diameter) × 21 cm (depth)) for further cultivation. Thirty pots of *T. chinensis* seedlings were planted in total (Figure S1B). The culture medium was vermiculite, sand and nutrient soil (1:1:1), and the plants were watered with 1/2 Hoagland nutrient solution every 5 days. After one month, seedlings were treated with salt stress at an initial concentration with 1/2 Hoagland nutrient solution containing 50 mmol/L NaCl, which was increased on successive days until the final NaCl concentration reached 200 mmol/L; thereafter, the treatment solution was applied every 5 days [12]. 

To assess the dynamic response of phyllosphere microorganisms of *T. chinensis* to salt secretion, we sampled six plants as one sample (approximately 10 g leaves per sample) before salt treatment, and eight phyllosphere samples were taken in total [56]. At the same time, nine rhizosphere soil samples were taken and sequenced as controls (CK). After 20 days of salt treatment, salt secretion was observed in *T. chinensis* leaves, and the sampling of the phyllosphere and rhizosphere was performed again for the salt stress group (SS) (Figure S1C).

### 4.2. Determination of Leaf Physiological Indexes and Soil Physicochemical Properties

The phyllosphere suspension mixes were divided into two parts, one for phyllosphere physicochemical characterization and the other for DNA extraction. According to the method of Pan et al. [12], the electrical conductivities (ECs) of the phyllosphere suspension mixes were determined using a conductivity meter. In addition, the rhizosphere soil was sampled at a depth of 10 cm. The root, stem, and leaf samples were washed with distilled water and dried in the oven (80 °C) to a constant weight, and the Na^+^ and K^+^ contents of the root, stem, leaf and phyllosphere were determined using flame spectrophotometry. The soil water content (SWC) was determined using the weighing method, and soil pH and electrical conductivity (EC) were determined at a soil–water ratio of 1:2.5. Soil total organic carbon (TOC) content was determined through potassium dichromate oxidation spectrophotometry. The sieved soil was introduced into a carbon and nitrogen analyzer for the determination of TOC and nitrogen (TN). The content of total phosphorus (TP) in the soil was determined using the sulfate–molybdenum dysprosium resistance colorimetric method. Soil Na^+^ and K^+^ contents were also determined by flame spectrophotometry [57].

### 4.3. Microbial DNA Extraction and PCR Amplification

Phyllosphere and rhizosphere DNA was extracted from screened soils using a 16-alkyltrimethylammonium bromide kit. Primers 341F (5′-CCTA YGGG RBGC ASCAG-3′) and 806R (5′-GGAC TACN NGGG TATC TAAT-3′) were used to amplify the V3 and V4 regions of the bacterial 16S rRNA gene in 54 qualified samples. The ITS1-2 gene regions of 54 eligible samples were amplified with primers ITS1 (5′-CTTG GTCA TTTA GAGG AAGT AA-3′) and ITS2 (5′-GCTG CGTT CTTC ATCG ATGC-3′). After PCR amplification, the Illumina NovaSeq PE250 (San Diego, CA, USA) platform was used for sequencing. Then, the OTUs and Shannon and ACE indices for the fungal communities were calculated using QIIME (V1.9.1) software [37,58]. 

### 4.4. Data Analysis

The phyllosphere and rhizosphere physicochemical characteristics of the 16 samples were statistically analyzed using ANOVA (Table 1). Redundancy analysis (RDA) was applied to distinguish the soil physicochemical properties, and the Na^+^ and K^+^ contents in the rhizosphere, tissues and phyllosphere under salt stress (Figure 1). Then, the Shannon and ACE indices were calculated using QIIME (V1.9.1) software [59] (Figure 2). Meanwhile, Spearman’s correlation analysis was performed between the Shannon and ACE indices, and physicochemical properties were inferred (Figure 2). The phyla (top 10 in terms of relative abundance) of the microbial community were analyzed using the “ggalluvial” package in R software (4.1) (Figure 3) [7]. In order to clarify the responses of the phyllosphere and rhizosphere microbial communities to salt stress, we determined the relationship between dominant phyla (relative abundances > 1%) and environmental factors via Spearman’s analysis (Figure 4) [33]. 

In this study, the correlation of bacterial and fungal OTUs was subjected to Spearman’s analysis (|r| < 0.9 and *p* < 0.01). In the calculation of the network, there were eight replicates in each group, and only seven OTUs that were not 0 were selected for correlation calculations out of the eight samples. After obtaining the correlation matrix data, the data were visualized using Cytoscape software (3.7.1) (Figure 5) [60]. After that, sub-network data such as node, edge, average density, transitivity, diameter, and average path length were calculated for each sample using the “igraph” package [61,62]. The PCA1 values were further obtained by principal component analysis of the sub-network data to characterize the complexity of the fungal network [63], where diameter and average path length were calculated via the inverse form (X-1). Spearman’s correlation analysis of NCI, subnetwork data, and environmental factors was also demonstrated for each sample plot. We further identified environmental factors with high explanatory rates for NCI via random forest analysis [64] (Figure 4).

Based on the functional annotation of prokaryotic taxa, energy, carbon cycle, nitrogen cycle, and sulfur cycle groups were distinguished [65]. Then, according to FUNGuild fungal function prediction, we classified the fungal functional guilds into symbiotrophic, saprotrophic, pathotrophic, arbuscular mycorrhizal (AM) and ectomycorrhizal (EcM) [66]. The correlation matriices of environmental factors, Shannon and ACE indices, and dominant phyla (average relative abundance > 1%) were calculated for both the phyllosphere and rhizosphere using the R software (4.1) “linkET” package. At last, the Mantel test was used to describe the correlation between microbial functional groups and environmental factors (Figure 5) [67,68].

## 5. Conclusions

Predicting individual plant/community–environment feedback effects through changes in microbial community structure and function remains a key challenge. In this study, we focused on the structural and functional characteristics of the phyllosphere microbial community of halophytes in response to salt secretion by performing controlled indoor experiments. Salt stress not only increased the soil EC, Na^+^ and K^+^ contents, but also increased the Na^+^ and K^+^ contents in different tissues of the plant, as well as in the phyllosphere. Not only that, but salt stress also reduced soil TOC, TN, and TP contents. In the phyllosphere, salt stress reduced the diversity indices of bacterial and fungal communities, and the relative abundance of Actinobacteriota, Basidiomycota, and Mortierellomycota decreased, while the relative abundance of Verrucomicrobiota and Ascomycota increased. In the phyllosphere’s bacteria–fungus co-occurrence network, salinity increased the network complexity, and random forest analysis has shown that EC, Na^+^ and K^+^ content were important influencing factors. Based on the functional prediction of bacteria and fungi, salt stress increased the relative abundances of phyllosphere bacteria energy sources and fungal pathotrophic groups, and decreased the relative abundance of saprotrophic groups. In addition, salt stress significantly decreased the rhizosphere bacterial community’s richness index, while, on the contrary, it increased the fungal community richness. The relative abundance of Actinobacteriota and Acidobacteriota showed a decreasing trend as affected by salinity, and the relative abundances of Bacteroidota, Verrucomicrobiota and Mortierellomycota increased significantly in the rhizosphere. The network complexity of the rhizosphere co-occurrence network was higher than that of the control network due to salinity, and Na^+^ and EC were the most important influencing factors. Salt stress also increased the relative abundance of functional groups of the carbon and phosphorus cycles of rhizosphere bacteria, and the functional groups were significantly correlated with EC and Na^+^. To the best of our knowledge, this is the first study to analyze the changes in the survival strategies of phyllosphere microbes associated with salt-secreting halophytes. This should be extended to field observations in future studies, so as to improve our understanding of halophyte–microbial community interactions in nature.

## Figures and Tables

**Table 1:** Physicochemical properties of rhizosphere soil and phyllosphere of *T. chinensis* under salt stress.

Physicochemical Properties	CK	SS
Ph. EC (µs/cm)	214.13 ± 15.37 b	811.38 ± 33.15 a
Ph-Na^+^ (mg/kg)	14.06 ± 1.97 b	464.38 ± 60.86 a
Ph-K^+^ (mg/kg)	2.45 ± 0.26 b	16.20 ± 1.32 a
Rh. pH	8.62 ± 0.03 b	9.10 ± 0.08 a
Rh. SWC	22.26 ± 1.95 a	22.91 ± 1.27 a
Rh. EC (µs/cm)	144.04 ± 6.99 b	2411.25 ± 422.03 a
Rh. TOC (g/kg)	26.68 ± 1.11 a	21.81 ± 0.73 b
Rh. TN (g/kg)	1.31 ± 0.09 a	0.98 ± 0.05 a
Rh. TP (g/kg)	0.55 ± 0.02 a	0.46 ± 0.02 a
Rh. Na^+^ (mg/kg)	89.04 ± 4.23 b	459.02 ± 50.56 a
Rh. K^+^ (mg/kg)	32.06 ± 2.84 a	30.32 ± 2.53 a
Root. Na^+^ (mg/kg)	54.26 ± 8.47 b	385.67 ± 21.25 a
Root. K^+^ (mg/kg)	16.32 ± 2.35 b	47.27 ± 4.11 a
Stem. Na^+^ (mg/kg)	23.92 ± 3.01 b	342.37 ± 19.33 a
Stem. K^+^ (mg/kg)	10.82 ± 1.29 b	41.62 ± 3.66 a
Leaf. Na^+^ (mg/kg)	13.60 ± 1.25 b	480.28 ± 97.56 a
Leaf. K^+^ (mg/kg)	8.38 ± 0.61 b	86.71 ± 6.42 a

Note: Ph.—phyllosphere; Rh.—rhizosphere. Different lowercase letters indicate differences between groups.

**Table 2:** Network topological features of rhizosphere soil and phyllosphere of *T. chinensis* under salt stress.

Topological Features	Ph. CK	Ph. SS	Rh. CK	Rh. SS
Nodes	175	280	1384	1150
Total edges	247	399	7531	6394
Positive edges	217	238	4196	5208
Negative edges	30	161	3335	1186
Modularity	0.846113	0.859674	0.574697	0.645207
Average path length	4.163033	8.326226	5.49876	5.181075
Graph diameter	12.89363	22.15457	16.57193	13.75342
Graph density	0.009889	0.00657	0.007581	0.008993
Clustering coefficient	0.490975	0.399516	0.436952	0.428124
Betweenness centralization	0.026054	0.102263	0.018319	0.023204
Degree centralization	0.043922	0.019292	0.042099	0.031276

Note: Ph.—phyllosphere; Rh.—rhizosphere.

**Table 3:** Relative abundances of bacterial functional groups in phyllosphere and rhizosphere of *T. chinensis* under salt stress.

Functional Groups	Ph. CK	Ph. SS	Rh. CK	Rh. SS
Energy source	20.44 ± 2.88 b	37.17 ± 1.91 a	23.34 ± 1.18 a	25.46 ± 1.95 a
C cycle	5.03 ± 0.49 a	3.25 ± 1.81 a	5.22 ± 0.69 b	11.39 ± 1.33 a
N cycle	44.70 ± 3.52 a	45.07 ± 4.41 a	4.50 ± 0.38 a	6.36 ± 1.12 a
P cycle	0.05 ± 0.02 a	0.22 ± 0.15 a	0.14 ± 0.04 b	1.27 ± 0.52 a

Note: Ph.—phyllosphere; Rh.—rhizosphere. Different lowercase letters indicate differences between groups.

**Table 4:** Relative abundance of fungal functional groups in phyllosphere and rhizosphere of *T. chinensis* under salt stress.

Functional Groups	Ph. CK	Ph. SS	Rh. CK	Rh. SS
Symbiotrophic	2.14 ± 0.94 a	0.23 ± 0.05 a	2.41 ± 0.86 a	1.34 ± 0.29 a
Saprotrophic	33.21 ± 8.36 a	3.34 ± 0.65 b	17.39 ± 3.85 a	21.58 ± 2.46 a
Pathotrophic	30.84 ± 8.83 b	70.68 ± 3.74 a	41.19 ± 8.33 a	44.61 ± 4.70 a
AM	0.71 ± 0.32 a	0.07 ± 0.01 a	0.71 ± 0.24 a	0.34 ± 0.14 a
EcM	1.28 ± 0.78 a	0.13 ± 0.04 a	0.97 ± 0.77 a	0.30 ± 0.13 a

Note: Ph.—phyllosphere; Rh.—rhizosphere. Different lowercase letters indicate differences between groups.

## Data Availability

The datasets presented in this study can be found in online repositories. The names of the repository/repositories and accession numbers can be found at: https://www.ncbi.nlm.nih.gov/, PRJNA925331 and PRJNA925432.

## Supplementary Materials

The following supporting information can be downloaded at: https://www.mdpi.com/article/10.3390/plants13081091/s1, Table S1: Relative abundance of phyllosphere and rhizosphere bacteria phylum in *T. chinensis* under salt stress; Table S2: Relative abundance of phyllosphere and rhizosphere fungi phylum in *T. chinensis* under salt stress; Table S3: Relative abundance of energy source functional groups of phyllosphere and rhizosphere bacteria in *T. chinensis* under salt stress; Table S4: Relative abundance of carbon cycle functional groups of phyllosphere and rhizosphere bacteria in *T. chinensis* under salt stress; Table S5: Relative abundance of nitrogen cycle functional groups of phyllosphere and rhizosphere bacteria in *T. chinensis* under salt stress; Table S6: Relative abundance of phosphorus cycle functional groups of phyllosphere and rhizosphere bacteria in *T. chinensis* under salt stress; Figure S1: Field growth (A) and salt treatment (B and C) of *T. chinensis*; Figure S2: Relative abundance (top 10) of phyllosphere and rhizosphere bacterial (A and B) and fungal (C and D) communities at phylum level of *T. chinensis* under salt stress.
