# Supplementary material for: Response of Phyllosphere and Rhizosphere Microbial Communities to Salt Stress of Tamarix chinensis
Source: Plants (Basel). 2024 Apr 13;13(8):1091. doi: 10.3390/plants13081091 (PMC11054833; doi:10.3390/plants13081091)
Supplement: Supplementary file 1 [file plants-13-01091-s001.zip › Supplementary Figures.pdf]

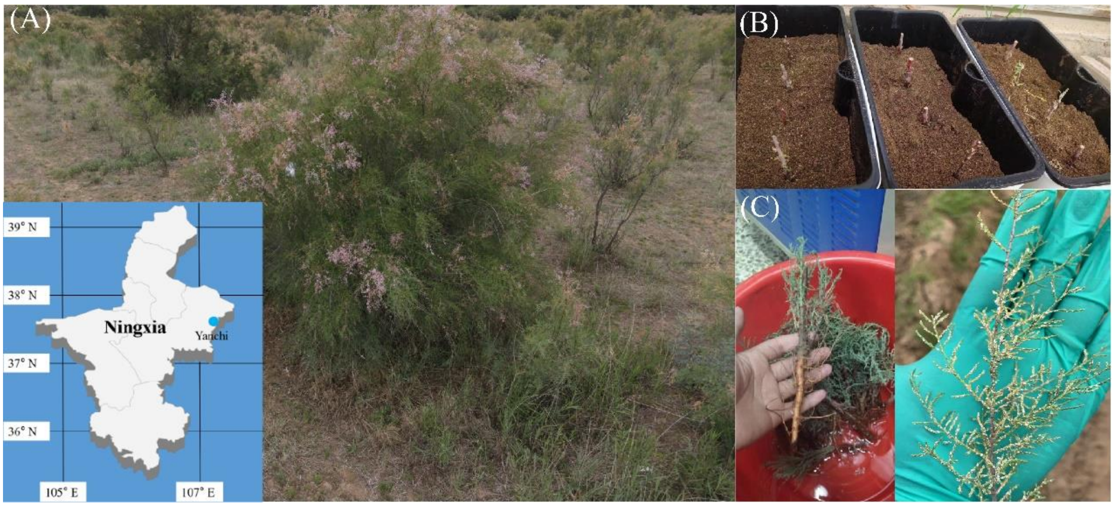

Figure S1. Field growth (A) and salt treatment (B and C) of T. chinensis.

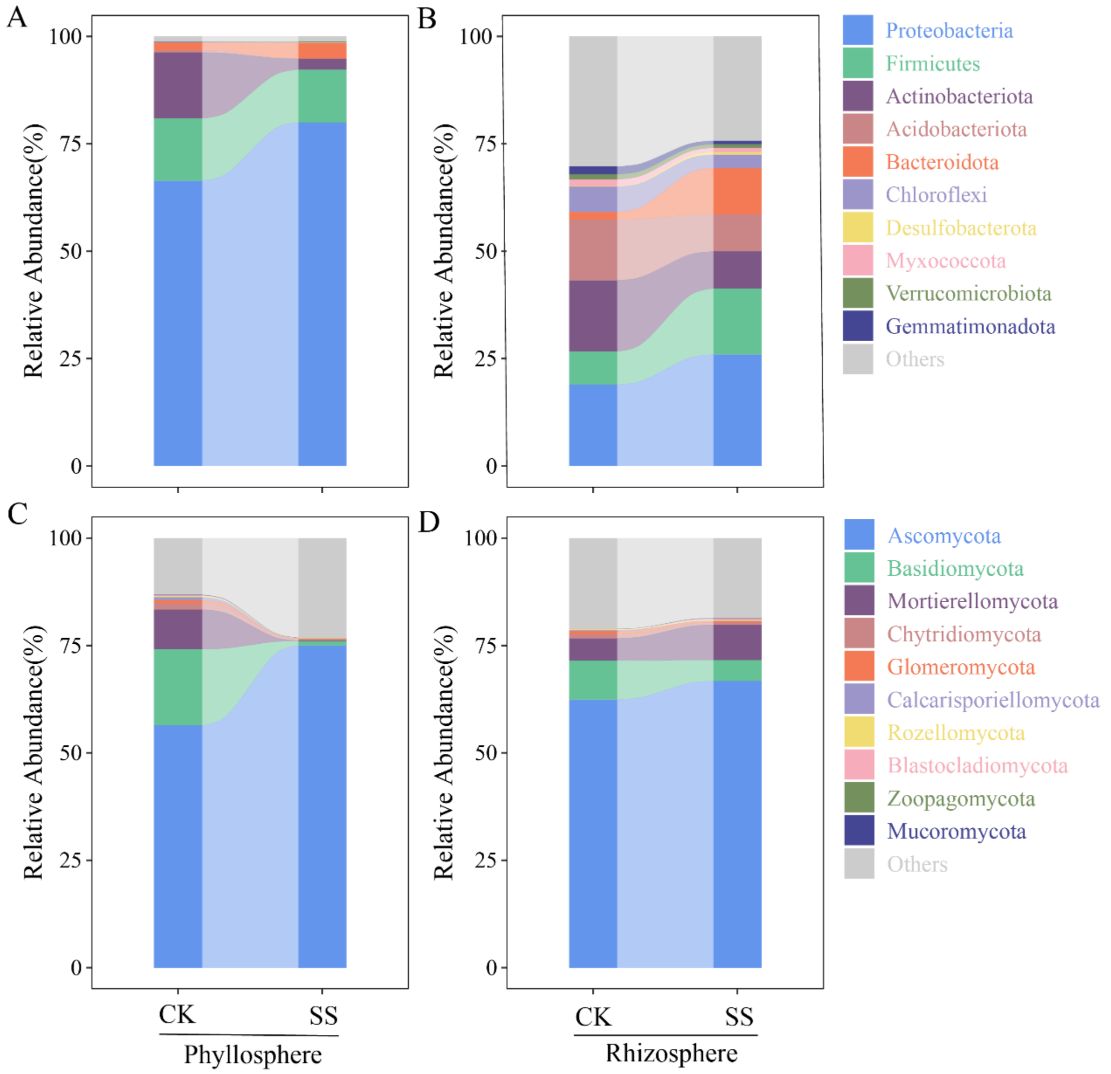

Figure S2. Relative abundance (top 10) of phyllosphere and rhizosphere bacterial (A and B) and fungal (C and D) communities at phylum level of T. chinensis under salt stress.
