# Supplementary material for: Response of Phyllosphere and Rhizosphere Microbial Communities to Salt Stress of Tamarix chinensis
Source: Plants (Basel). 2024 Apr 13;13(8):1091. doi: 10.3390/plants13081091 (PMC11054833; doi:10.3390/plants13081091)
Supplement: Supplementary file 1 [file plants-13-01091-s001.zip › Supplementary Tables.pdf]

Table S1. Relative abundance of phyllosphere and rhizosphere bacteria phylum in *T. chinensis* under salt stress

Functional groups	Ph. CK	Ph. SS	Rh. CK	Rh. SS
Proteobacteria	0.66±0.06a	0.80±0.09a	0.19±0.01b	0.26±0.02b
Firmicutes	0.14±0.05a	0.12±0.07a	0.08±0.02a	0.015±0.04a
Actinobacteriota	0.15±0.02a	0.03±0.01c	0.17±0.01a	0.09±0.01b
Acidobacteriota	0.0047±0.002c	0.0002±0.00008c	0.1412±0.0136a	0.0859±0.0148b
Bacteroidota	0.02±0.004b	0.04±0.02b	0.02±0.001b	0.11±0.03a
Chloroflexi	0.0003±0.00008c	0.0004±0.0001c	0.0590±0.0003a	0.0313±0.0032b
Desulfobacterota	0.0004±0.0002a	0.0003±0.00003a	0.0017±0.0004a	0.0057±0.0041a
Myxococcota	0.0004±0.0002c	0.0010±0.0007c	0.0152±0.0005a	0.0097±0.0017b
Verrucomicrobiota	0.0003±0.00009b	0.0013±0.0002a	0.0119±0.0016b	0.0087±0.0033a
Gemmatimonadota	0.0003±0.0001c	0.0001±0.00003c	0.0184±0.0009a	0.0080±0.0016b

Note: Ph: phyllosphere; Rh: rhizosphere. Different lowercase letters indicate differences between groups.

Table S2. Relative abundance of phyllosphere and rhizosphere fungi phylum in *T. chinensis* under salt stress

Functional groups	Ph. CK	Ph. SS	Rh. CK	Rh. SS
Ascomycota	0.56±0.08b	0.75±0.04a	0.62±0.06ab	0.67±0.04ab
Basidiomycota	0.18±0.09a	0.01±0.001b	0.09±0.02ab	0.05±0.005ab
Mortierellomycota	0.093±0.021a	0.001±0.0003c	0.052±0.004b	0.082±0.013ab
Chytridiomycota	0.014±0.007a	0.005±0.002a	0.009±0.004a	0.005±0.002a

Glomeromycota	0.007±0.003a	0.0006±0.0001a	0.007±0.002a	0.003±0.001a
Calcarisporiellomycota	0.0076±0.0071a	0	0.0025±0.0019a	0.0005±0.0003a
Rozellomycota	0.0029±0.0014a	0.0007±0.0001a	0.0019±0.0006a	0.0014±0.0003a
Blastocladiomycota	0.0009±0.0003ab	0.00004±0.00002c	0.0001±0.00007b	0.0032±0.0017a
Zoopagomycota	0.0006±0.0004a	0.00007±0.00004a	0.008±0.004a	0.0013±0.0008a
Mucoromycota	0.0011±0.0004a	0.0008±0.0004a	0.00007±0.00004a	0.0007±0.0004a

Note: Ph: phyllosphere; Rh: rhizosphere. Different lowercase letters indicate differences between groups.

Table S3. Relative abundance of energy source functional groups of phyllosphere and rhizosphere bacteria in *T. chinensis* under salt stress

Functional groups	Ph. CK	Ph. SS	Rh. CK	Rh. SS
chemoheterotrophy	0.11±0.01b	0.20±0.004a	0.14±0.006b	0.17±0.01a
aerobic chemoheterotrophy	0.09±0.01b	0.17±0.02a	0.09±0.008b	0.08±0.009b
photoautotrophy	0.000062±0.000032a	0.000934±0.000887a	0.001276±0.000339a	0.001994±0.001804a
phototrophy	0.0001±0.00005a	0.0018±0.0008a	0.0014±0.0003a	0.0022±0.001a
photoheterotrophy	0.000047±0.000027b	0.000854±0.000235a	0.000134±0.000038b	0.000229±0.000149b
oxygenic photoautotrophy	0.000062±0.000032a	0.000934±0.000887a	0.001276±0.000339a	0.001982±0.000179a

Note: Ph: phyllosphere; Rh: rhizosphere. Different lowercase letters indicate differences between groups.

Table S4. Relative abundance of carbon cycle functional groups of phyllosphere and rhizosphere bacteria in *T. chinensis* under salt stress

Functional groups	Ph. CK	Ph. SS	Rh. CK	Rh. SS
fermentation	0.03±0.005b	0.03±0.02b	0.04±0.007b	0.09±0.01a
xylanolysis	0.0003±0.0002b	0.0021±0.0018ab	0.0009±0.0005b	0.0062±0.002b
methylotrophy	0.0069±0.0019a	0.0004±0.00005b	0.0007±0.00006b	0.0005±0.0001b
chitinolysis	0.0012±0.0008ab	0.00003±0.000007b	0.0007±0.000008ab	0.0027±0.0012a
cellulolysis	0.0011±0.0005ab	0.0002±0.0001b	0.0016±0.0006a	0.0011±0.0002ab
ligninolysis	0.000044±0.000009b	0	0.000081±0.000011a	0.000068±0.000023a
methanol oxidation	0.0069±0.0019a	0.0004±0.00005b	0.0007±0.00006b	0.0004±0.00008b
methanotrophy	0	0.000002±0.000001a	0.000014±0.000009a	0.000092±0.000013a
aliphatic non methane hydrocarbon degradation	0.000057±0.000024b	0.000001±0.0000008b	0.0001113±0.000019ab	0.00025±0.000104a
hydrocarbon degradation	0.0002±0.0005b	0.0011±0.0007b	0.0002±0.000005b	0.0124±0.0064a
aromatic hydrocarbon degradation	0.0001±0.00003a	0.0011±0.0007a	0.0002±0.00004a	0.0004±0.0001a
aromatic compound degradation	0.0006±0.0001c	0.0013±0.0007bc	0.0052±0.0004a	0.0021±0.0003b

Note: Ph: phyllosphere; Rh: rhizosphere. Different lowercase letters indicate differences between groups.

Table S5. Relative abundance of nitrogen cycle functional groups of phyllosphere and rhizosphere bacteria in *T. chinensis* under salt stress

Functional groups	Ph. CK	Ph. SS	Rh. CK	Rh. SS
ureolysis	0.2898±0.05429a	0.0002±0.00003b	0.0027±0.0007b	0.0016±0.0002b
nitrate reduction	0.05±0.01b	0.15±0.01a	0.013±0.001c	0.018±0.003c
nitrogen respiration	0.037±0.016b	0.14±0.016a	0.003±0.0005b	0.011±0.002b
nitrate respiration	0.037±0.0162b	0.145±0.0157a	0.003±0.0005b	0.011±0.002b
nitrite respiration	0.0096±0.0019a	0.0042±0.0010b	0.0008±0.00008c	0.0047±0.0011b
aerobic ammonia oxidation	0	0.000006±0.000003b	0.009236±0.002173a	0.005149±0.001933a
nitrite denitrification	0.0074±0.0021a	0.0032±0.0008b	0.0006±0.00007b	0.0005±0.0001b
nitrate denitrification	0.0074±0.0021a	0.0032±0.0008b	0.0006±0.00007b	0.0005±0.0001b
nitrification	0	0.000006±0.000003b	0.009236±0.002173a	0.005156±0.001931a
nitrite ammonification	0.0022±0.0008ab	0.0011±0.00007b	0.0002±0.0008b	0.0043±0.0011a
nitrogen fixation	0.000206±0.000102c	0.000076±0.000038c	0.001826±0.000375a	0.000942±0.000298b
nitrate ammonification	0.001619±0.000809a	0.000015±0.000008b	0.000052±0.000014b	0.00062±0.000183ab

Note: Ph: phyllosphere; Rh: rhizosphere.

Table S6. Relative abundance of phosphorus cycle functional groups of phyllosphere and rhizosphere bacteria in *T. chinensis* under salt stress

Functional groups	Ph. CK	Ph. SS	Rh. CK	Rh. SS
sulfate respiration	0.000155±0.000114b	0.000051±0.000013b	0.000542±0.000192b	0.000927±0.000342a
sulfite respiration	0	0.000003±0.000002a	0.000004±0.000004a	0.000181±0.000144a
sulfur respiration	0	0	0	0.0001±0.000008a
respiration of sulfur compounds	0.000155±0.000114b	0.000068±0.000019b	0.000575±0.000197b	0.005884±0.002647a
thiosulfate respiration	0	0.000018±0.000011b	0.000033±0.000026b	0.004881±0.002803a
dark sulfide oxidation	0.000012±0.000006a	0.000011±0.000004a	0.000020±0.000011a	0.000103±0.000012a
dark sulfur oxidation	0.000006±0.000004a	0	0	0.000094±0.000087a
dark sulfite oxidation	0	0	0	0.000095±0.000082a
dark oxidation of sulfur compounds	0.000089±0.000039a	0.001049±0.00077a	0.000104±0.000044a	0.000324±0.000206a
dark thiosulfate oxidation	0.000083±0.000037a	0.001038±0.000772a	0.000084±0.000039	0.000127±0.000101a

Note: Ph: phyllosphere; Rh: rhizosphere.
